# Characterizing building blocks of resource constrained biological networks

**DOI:** 10.1186/s12859-019-2838-x

**Published:** 2019-06-20

**Authors:** Yuanfang Ren, Ahmet Ay, Alin Dobra, Tamer Kahveci

**Affiliations:** 1 Computer and Information Science and Engineering, University of Florida, Gainesville, 32611 FL USA; 2 Departments of Biology and Mathematics, Colgate University, Hamilton, 13346 NY USA

**Keywords:** Biological networks, Network motif, Partial overlap

## Abstract

**Background:**

Identification of motifs–recurrent and statistically significant patterns–in biological networks is the key to understand the design principles, and to infer governing mechanisms of biological systems. This, however, is a computationally challenging task. This task is further complicated as biological interactions depend on limited resources, i.e., a reaction takes place if the reactant molecule concentrations are above a certain threshold level. This biochemical property implies that network edges can participate in a limited number of motifs simultaneously. Existing motif counting methods ignore this problem. This simplification often leads to inaccurate motif counts (over- or under-estimates), and thus, wrong biological interpretations.

**Results:**

In this paper, we develop a novel motif counting algorithm, *Partially Overlapping MOtif Counting* (*POMOC*), that considers capacity levels for all interactions in counting motifs.

**Conclusions:**

Our experiments on real and synthetic networks demonstrate that motif count using the *POMOC* method significantly differs from the existing motif counting approaches, and our method extends to large-scale biological networks in practical time. Our results also show that our method makes it possible to characterize the impact of different stress factors on cell’s organization of network. In this regard, analysis of a *S. cerevisiae* transcriptional regulatory network using our method shows that oxidative stress is more disruptive to organization and abundance of motifs in this network than mutations of individual genes. Our analysis also suggests that by focusing on the edges that lead to variation in motif counts, our method can be used to find important genes, and to reveal subtle topological and functional differences of the biological networks under different cell states.

## Background

Biological networks describe interactions between molecules such as genes and proteins [1, 2]. These networks are often modeled as graphs where nodes and edges represent molecules and their interactions, respectively. Biological networks are involved in many key biological processes including transcriptional regulation, interactions between a cell and its environment, and controlling a cell’s specificity [3–5]. Understanding biological networks is essential for understanding how cells function. Efforts on computational analysis of biological networks have been growing rapidly in recent years as large-scale data collection at low cost is now possible.

**Fig. 1:**
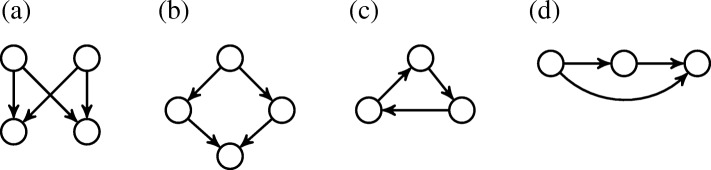
Four conserved motifs studied frequently in the literature. (**a**) Bifan. (**b**) Biparallel (**c**) Cascade (**d**) Feed forward

One of the most fundamental challenges in computational network studies is the motif counting problem. A network motif is a pattern of local interconnections (i.e., a small subnetwork) observed significantly more frequently in a given network than in a random network of the same size [6, 7]. Existing studies have already uncovered existence of network motifs such as feed forward loop and bifan (see Fig. 1) [8, 9]. Motifs utilize the basic control mechanisms to govern biologically important dynamic behaviors, such as oscillations, generation of molecular pulses, and rapid or delayed responses [7, 10]. Thus, the presence or relative abundance of motifs in biological networks is often used to characterize their topology, function, and robustness [11, 12]. Network motifs have been effectively used to study the biological processes that regulate transcription [9], to find the genetic factors that impact various diseases [13, 14] and to discover new drugs [15].

**Fig. 2:**
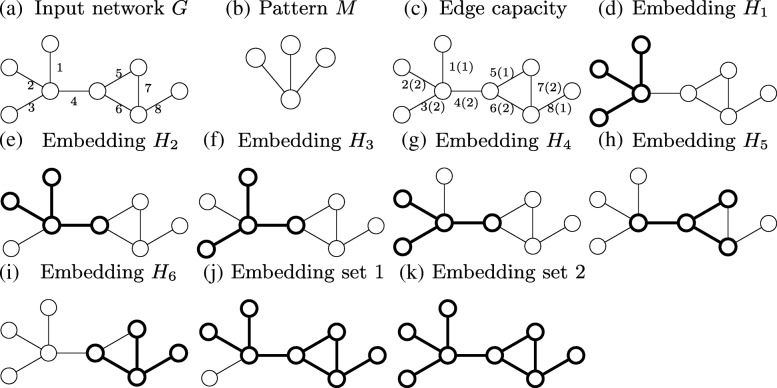
A hypothetical network and its embeddings of a given pattern. (**a**) A network with eight nodes and eight edges. (**b**) A motif pattern. (**c**) A network with edge capacities. *x*(*y*) denotes the edge *x* has the capacity *y*. (**d**) - (**i**) Six motif embeddings in the network. (**j**) An embedding set includes embeddings *H*_2_,*H*_5_ and *H*_6_. (**k**) An embedding set includes embeddings *H*_1_,*H*_4_,*H*_5_ and *H*_6_

Identifying motifs and counting them in biological networks is a computationally challenging task as it requires solving the subgraph isomorphism problem, which is NP-complete [16]. Several methods have been developed to count instances of a motif in a given network [17–20]. These methods could be categorized into two classes [17]. The first class counts all instances of a given motif ignoring the fact that some motifs may share edges (*F*_1_ measure) [19]. The second class counts all non-overlapping instances of a given motif, i.e., those which do not share any edge (*F*_2_ measure). Figure 2 shows the difference between these two frequency measures on a hypothetical network *G*. Consider the motif pattern *M* in Fig. 2b. Our input network *G* in Fig. 2a yields six possible embeddings of *M* shown in Fig. 2d-i. Thus, *F*_1_ measure of *M* in *G* is six. However, out of these six embeddings at most two can be chosen without picking the same edge multiple times (e.g., Fig. 2d and i). Thus, *F*_2_ measure of *M* in *G* is two.

Notice that both *F*_1_ and *F*_2_ measures make opposite, yet very strong assumptions regarding how the cells realize interactions. The former one assumes that the same interaction can participate in an arbitrary number of motif instances at the same time. The latter one limits the participation of an interaction to a single motif instance. Although, these two assumptions simplify the motif counting problem, they rarely reflect how cells operate interactions. Each interaction utilizes the molecules participating in that interaction. Thus the abundance of the interacting molecules makes it possible to include the corresponding edges appear in multiple (yet limited) number of motif instances. For example, depending on the concentration level of a metabolite, an enzymatic reaction may take place in one (low reaction/edge capacity) or many metabolic pathways (high edge capacity) [21–23]. We use the term *capacity* of an edge to denote the number of motif instances that edge can participate simultaneously. Thus, even if two cells have the same underlying biological network topology, they may yield different number of motifs of the same topology. For example, if we allow partial overlap of the motif *M* in the network *G* (see Fig. 2b and c), we find four possible embeddings of motif *M* in *G* (Fig. 2d, g, h and i). *A motif counting approach that ignores the edge capacities will lead to unrealistic motif counts, and wrong biological interpretations. Thus, motif counting approaches that take edge capacities into account are needed.*

In the literature, several approaches have been developed to count motifs while also incorporating edge information [24–27]. These methods are designed for weighted networks, and find motifs with specific weights. As we explain in the subsequent sections in detail, the problem considered in this paper is fundamentally different (See “Discussion and Conclusions” section for details on motifs in weighted networks).

**Our contributions.** In this study, we build a new motif counting algorithm that allows partial overlap between different embeddings of a given motif on the target network. Briefly, given a target network, a motif topology, and a positive capacity value for each interaction in the target network, we count the maximum number of ways to place the motif on the target network, so that no edge appears in more motif embeddings than its capacity. Notice that the classical counting measures *F*_1_ and *F*_2_ are special instances of our measure [28, 29]. If we set the capacity of all edges to one, our motif counting problem reduces to non-overlapping motif counting with *F*_2_ measure. Similarly, if we set the edge capacities to infinity our problem reduces to motif counting using *F*_1_ measure.

We develop a novel motif counting method called *Partially Overlapping MOtif Counting* (*POMOC*), that computes the number of partially overlapping instances of a given motif in a given network. *POMOC* algorithm first finds all instances of a given motif *M* in the network *G*, then it chooses the motif instances that are guaranteed to exist without using any edge more than its capacity. For a given motif embedding, if the capacities of its edges are more than the number of embeddings those edges are part of, this embedding exists in our solution. Next, for the remaining motif instances, our algorithm randomly adds some embeddings whose edges are not more than the capacity constraints, into the resulting embedding set. It gradually expands the resulting set by taking one embedding out of the resulting set and inserting another two embeddings to the set.

Our experimental results on synthetic and real datasets demonstrate that our algorithm finds vastly different motif counts than *F*_1_ and *F*_2_ measures. Since biological interactions are resource limited, leading to varying edge capacities, we hypothesize that our method provides a more accurate approach to counting network motifs in biological networks than existing methods. Although, the *POMOC* method is slightly slower than motif counting with *F*_1_ and *F*_2_ measures, our method remains to be practical for all network sizes and motifs we test here. Our results on a *S. cerevisiae* transcriptional regulatory network suggest that oxidative stress is more disruptive to abundance and organization of network motifs than genetic mutations. Our analysis on the yeast network also suggests that our method can be used to find the key genes, which lead to topological and functional differences in biological networks under varying genetic backgrounds and growth conditions.

The rest of the paper is organized as follows. We present our algorithm in “Methods” section. We experimentally evaluate our method in “Results” section and provide a brief conclusion in “Discussion and Conclusions” section.

## Methods

Here, we describe our method for counting partially overlapping motifs in networks. Preliminaries and problem definition section provides the preliminaries needed to describe our method. Counting partial overlapping motifs section discusses our algorithm.

### Preliminaries and problem definition

We denote a given biological network with graph *G*=(*V,E*,**c**), where *V*={*v*_1_,*v*_2_,…,*v*_*n*_} and *E*={*e*_1_,*e*_2_,…,*e*_*m*_} represent the set of nodes (molecules) and the set of edges (interactions) among those nodes, respectively. The function $\mathbf {c}:E \rightarrow \mathbb {Z}^{+}$ shows the capacity of the edges. To simplify our notation, in the rest of this paper, ∀*e*_*i*_∈*E* we use *c*_*i*_ to denote **c**(*e*_*i*_) (i.e., the capacity of the edge *e*_*i*_) and the vector *C*=(*c*_1_,*c*_2_,…,*c*_*m*_) to represent the capacity of all edges in *E* in sorted order of edge indices. For example, in Fig. 2c, the value in the form *x*(*y*) denotes that edge *x* has capacity *y*.

Given a motif pattern *M*, we represent the *i*th embedding of *M* in *G* with *H*_*i*_⊆*E* (i.e., *H*_*i*_ constitutes a subgraph of *G*, which is topologically isomorphic to *M*). We denote the set of all possible embeddings of *M* in *G* with $\mathcal {H}(M)$. Given an edge *e*_*i*_∈*E* and a subset $\mathcal {H}^{\prime }$ of $\mathcal {H}(M)$, we denote the set of all embeddings in $\mathcal {H}^{\prime }$ containing edge *e*_*i*_ with $\mathrm {f_{i}}(M,\mathcal {H}^{\prime }) = \{ H_{j} \in \mathcal {H}^{\prime }| e_{i} \in H_{j} \}$. We denote the number of embeddings in the set $\mathcal {H}^{\prime } \subseteq \mathcal {H}(M)$ containing each interaction in *E* with the vector $C_{\mathcal {H}^{\prime }} = (|\mathrm {f}_{1}(M,\mathcal {H}^{\prime })|, |\mathrm {f}_{2}(M,\mathcal {H}^{\prime })|, \dots, |\mathrm {f}_{m}(M,\mathcal {H}^{\prime })|)$. We say that $\mathcal {H}^{\prime }$ is *feasible* if no interaction appears in more embeddings in $\mathcal {H}^{\prime }$ than its capacity that is $\forall i, |\mathrm {f}_{i}(M,\mathcal {H}^{\prime })| \le c_{i}$. Figure 2b to i explain this on an example. Here, the capacity of the edges is *C*=(1,2,2,2,1,2,2,1). This network yields six embeddings (see Fig. 2d to i). Consider the subset $\mathcal {H}^{\prime }=\{H_{1},H_{6}\}$. The capacity $\mathcal {H}^{\prime }$ uses is $C_{\mathcal {H}^{\prime }} = (1,1,1,0,0,1,1,1)$, which is less than the imposed capacity constraints in *C*. Thus, $\mathcal {H}^{\prime }$ is feasible. The subset of embeddings {*H*_1_,*H*_2_}, however, is not feasible as this set contains edge *e*_1_ twice, which is more than its capacity (*c*_1_=1).

Next, we formally define the partially overlapping motif counting problem.

#### Definition 1.

(PARTIALLY OVERLAPPING MOTIF COUNTING). Consider a graph *G*=(*V,E*,**c**) conditioned with edge capacity constraints. Given a motif pattern *M*, partially overlapping motif counting problem seeks to find a largest feasible subset of the set of motif embeddings $\mathcal {H}(M)$.

Notice that Definition 1 provides a general formulation of motif counting problem. When the capacity constraints of each edge is set to infinity it reduces to a motif counting problem using *F*_1_ measure. When the capacity of all edges are set to one, it counts non-overlapping motifs (i.e., *F*_2_ measure). Next, we present the partially overlapping motif counting problem on an example.

#### Example 1.

Different ways to select embeddings leads to different number of possible embeddings. Consider the network in Fig. 2c with capacity constraints *C*=(1,2,2,2,1,2,2,1). Embedding set 1 (see Fig. 2j) includes embeddings *H*_2_,*H*_5_ and *H*_6_, and is feasible as its usage of the capacity is (1,1,0,2,1,2,1,1). Embedding set 2 (see Fig. 2k) includes embeddings *H*_1_,*H*_4_,*H*_5_ and *H*_6_. This set is also feasible. The number of embeddings in this set is four. The partially overlapping motif count is thus four as this is the largest set of feasible embeddings.

Counting partially overlapping motifs is an NP complete problem for several reasons. First, it requires solving the subgraph isomorphism problem, which is NP-complete [30]. Furthermore, as we explain above the *F*_2_ count is a special instance of the partially overlapping motif counting problem as we can reduce the non-overlapping motif counting problem to partially overlapping motif counting by setting the capacity of all edges to one. The Maximum Independent Set (MIS) problem, which is NP-complete [16], reduces to the non-overlapping motif counting problem [19, 29]. Thus the partially overlapping motif counting problem requires solving at least two NP-complete problems. In this paper, we develop a scalable method to tackle this problem using the local search strategy.

### Counting partial overlapping motifs

In this section, we discuss our *POMOC* algorithm. Algorithm 1 presents the pseudo-code of our method. Our algorithm takes a network *G*=(*V,E*,**c**) and a motif pattern *M* as input. Briefly, our algorithm has four main steps: (1) We locate all possible embeddings of *M* in *G* (line 1). At this step, we ignore the number of embeddings of *M* sharing each edge. (2) We determine the embeddings, which are guaranteed to exist in the final solution (lines 2-5). (3) We construct an initial, random yet feasible, solution by including a subset of the remaining embeddings in the set found in Step 2 (lines 5-6). (4) We iteratively improve this solution by replacing an embedding in the current solution with two or one new embeddings without violating feasibility of the solution (lines 7-11).

The first step of our algorithm is identical to computing the *F*_1_ count. This is a well studied problem in the literature. We use the method developed by Elhesha *et al* [31] for this step as it is one of the most recent and efficient methods. One can however replace this step with another method for *F*_1_ count without affecting the rest of our algorithm. Below, we explain Steps 2, 3, and 4 in detail.

**Step 2.** An embedding *H*_*r*_ is guaranteed to exist in the solution set if each edge of *H*_*r*_ has large enough capacity to realize all embeddings that have this edge. Formally, *H*_*r*_ exists in result set if $\forall e_{i} \in H_{r}, |\mathrm {f}_{i}(M,\mathcal {H}(M))|\le c_{i}$. For example, in the network in Fig. 2c, embedding *H*_6_ (Fig. 2i) satisfies this criteria. We prove the correctness of this step at the end of this section.

**Fig. 3:**
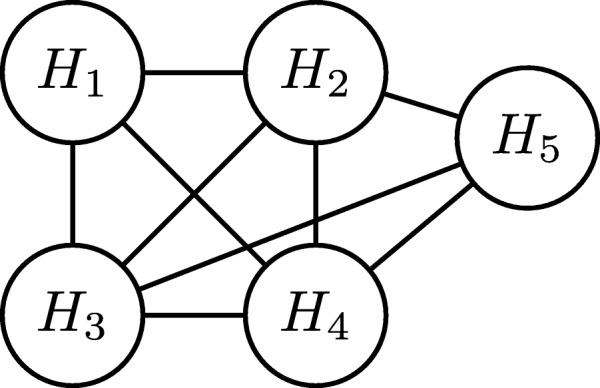
The overlap graph of the five embeddings in Fig. 2d-i


**Step 3.** Once we identify the set of all embeddings, which are guaranteed to be in the final solution, we move them from the set $\mathcal {H}$ into solution set $\mathcal {A}$. We then update the capacity constraints in the input graph *G* as follows. For each embedding $H_{r} \in \mathcal {A}$, we reduce the capacities of all the edges *e*_*i*_∈*H*_*r*_ by one as *H*_*r*_ is in the solution set. We then build a new graph, called the *overlap graph* for the remaining embeddings in $\mathcal {H}$. Each node in this graph corresponds to an embedding in $\mathcal {H}$. We include an edge between two nodes if their corresponding embeddings share at least one edge. Figure 3 depicts the overlap graph of the remaining five embeddings after moving embedding *H*_6_ from $\mathcal {H}$ into the result set. Next, we generate a random feasible solution iteratively using the overlap graph as follows. At each iteration, we randomly pick a node *v*_*r*_ from the overlap graph, and include the corresponding embedding (say *H*_*r*_) in the solution. We then reduce the capacity of all the edges of *H*_*r*_ in *G* by one. If the capacity of an edge drops to zero, it means that that edge cannot participate in any other embedding (say *H*_*s*_) without violating the feasibility of solution. If such an embedding *H*_*s*_ exists, its corresponding node in the overlap graph is a neighbor of *v*_*r*_ as they share that edge in *G*. Thus, we remove these neighbors of *v*_*r*_ in the overlap graph, which denote an embedding with an edge of zero capacity. We repeat these iterations to grow the random feasible solution set until the overlap graph becomes empty.

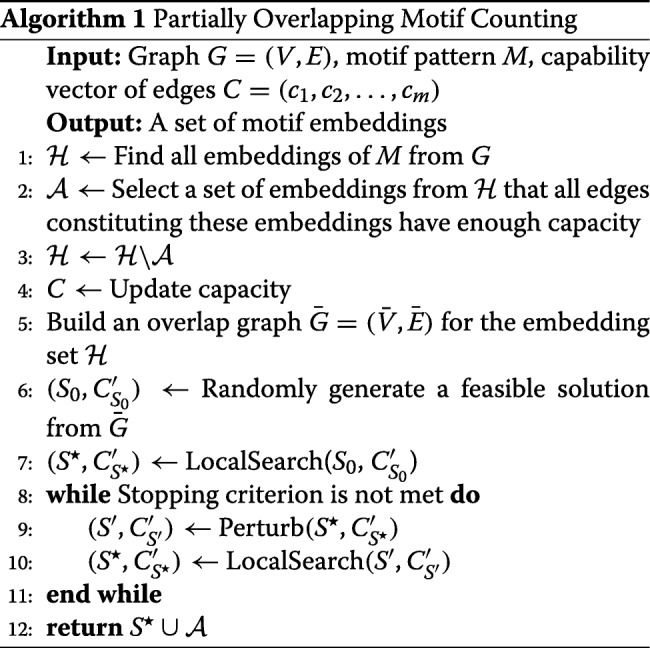


**Step 4.** So far, we have generated an initial feasible solution, which consists of those embeddings, which are guaranteed to be in the final result (Step 2) and those chosen randomly (Step 3). Next, we try to increase the size of this solution set iteratively by applying two strategies, namely *local search* and *perturbation*. We elaborate on these strategies next.

Local search iteratively increases the size of the solution set by replacing an embedding in the solution set with two other embeddings while maintaining the feasibility of the solution. For each embedding *H*_*i*_ in the solution, we study its corresponding node *v*_*i*_ in the overlap graph along with the set of neighboring nodes *v*_*i*_. We find all pairs of nodes (*v*_*j*_,*v*_*k*_) in the overlap graph, which satisfy all of the following two conditions.

(i) *v*_*j*_ and *v*_*k*_ are neighbors of *v*_*i*_.

(ii) Replacing the embedding *H*_*i*_ corresponding to *v*_*i*_ in the solution set with those of *H*_*j*_ and *H*_*k*_ corresponding to nodes *v*_*j*_ and *v*_*k*_ respectively does not violate the feasibility of the solution.

Once we identify all such node pairs, we randomly pick one and replace the embedding for node *v*_*i*_ with those of *v*_*j*_ and *v*_*k*_. After each swap, we update the capacity of the edges contained in the embeddings *H*_*i*_,*H*_*j*_ and *H*_*k*_. Notice that each swap operation increases the solution set size by one.

Similar to many local search algorithms, *POMOC* has the potential to get trapped in the local optimum. To escape the local optimum, before each local search, *POMOC* perturbs the current best solution to explore a slightly different search space. More specifically, *POMOC* first selects a node *v*_*i*_ such that *H*_*i*_ is in the solution set and replaces it with an embedding *H*_*j*_ such that *v*_*j*_ is a neighbor of *v*_*i*_ in the overlap graph if the solution set remains feasible. As a result, the size of the solution set remains unchanged after the perturbation. For each node in the solution, *POMOC* does a Bernoulli trial with a probability value *p* to determine if a replacement is needed for this node. Given a solution *S*, *POMOC* does |*S*|×*p* replacements approximately. If a replacement is decided, *POMOC* finds all valid non-solution neighbors and randomly pick one to replace. Here, valid neighbors means the replacement of these nodes is consistent with the capacity constraints.

We repeat updating the solution by applying local search and perturbation until the size of the solution set does not improve for user supplied number of iterations. Finally, we prove the correctness of Step 2 of our algorithm in the following theorem.

#### Theorem 1.

Given a graph *G*=(*V,E*,**c**) conditioned with edge capacity constraints and a motif pattern *M*, consider the set of all possible embeddings of *M*, $\mathcal {H}(M)$. An embedding $H_{r} \in \mathcal {H}(M)$ must be included by partially overlapping motif counting problem, if $\forall e_{i} \in H_{r}, |\mathrm {f}_{i}(M,\mathcal {H}(M))|\le c_{i}$.

**Proof.** We prove the theorem by contradiction. Assume that $\mathcal {H}^{\prime }$ is the largest feasible subset of the set of motif embeddings $\mathcal {H}(M)$, which does not have *H*_*r*_. That is, $\mathcal {H}^{\prime } \subseteq \mathcal {H}(M) \backslash H_{r}$. Now we construct a larger subset of embeddings $\mathcal {H}^{\prime \prime } = \mathcal {H}^{\prime } \cup \{H_{r}\}$. ∀*e*_*i*_∉*H*_*r*_, we have

$$|\mathrm{f}_{i}(M,\mathcal{H}^{\prime\prime})| = |\mathrm{f}_{i}(M,\mathcal{H}^{\prime})| \le c_{i} $$

In addition, ∀*e*_*i*_∈*H*_*r*_, we have

$$\begin{array}{*{20}l} |\mathrm{f}_{i}(M,\mathcal{H}^{\prime\prime})| =& |\mathrm{f}_{i}(M,\mathcal{H}^{\prime} \cup H_{r})|\\ \le & |\mathrm{f}_{i}(M,\mathcal{H}(M) \backslash H_{r} \cup H_{r})| \\ = & |\mathrm{f}_{i}(M,\mathcal{H}(M))|\le c_{i} \end{array} $$

Thus, $\mathcal {H}^{\prime \prime }$ is also a feasible subset of embeddings, which yields a contradiction to the assumption that $\mathcal {H}^{\prime }$ which does not include *H*_*r*_ is the largest feasible subset of $\mathcal {H}(M)$. Thus, an embedding *H*_*r*_ must be included by partially overlapping motif counting problem if $\forall e_{i} \in H_{r}, |\mathrm {f}_{i}(M,\mathcal {H}(M))|\le c_{i}$. □

## Results

In this section, we experimentally evaluate performance of our method on synthetic and real datasets. We consider four motif topologies, which are commonly studied in the literature; namely bifan, biparallel, cascade and feed forward loop (see Fig. 1). These motifs have been shown to be overrepresented in biological networks under the *F*_1_ count [8, 9]. We compare our method’s motif count and running time to two existing approaches: counting with *F*_1_ and *F*_2_ measures. First, we describe in detail the synthetic and real datasets used in our experiments. We then present the results.

### Datasets

SYNTHETIC DATASET. We generate directed random networks to test robustness and scalability of our algorithm under three network parameters: network size (i.e., number of nodes), edge capacity, and topology model. We generate synthetic networks of varying sizes and edge capacities using three network topology models: Erdos-Renyi random graph (ER) [32], Watts-Strogatz small-world (WS) [33] and Barabasi-Albert preferential attachment (BA) [34]. We randomly assign the direction of each edge. We set a capacity value to each edge using two alternative approaches. In the first approach, we set the capacity of all edges to the same value; 1, 2 or 3. In the second approach, we randomly assign each edge capacity to a value between 1 and 3, thus different edges can have different capacities.

REAL DATASET. We use *S. cerevisiae* transcriptional regulatory network [8, 35]. This network contains 690 nodes and 1081 edges. We use the *S. cerevisiae* gene expression dataset, *GSE26169*, obtained from the GEO database to set capacities of interactions [36]. This dataset contains expression data under control and oxidative stress conditions in seven genetic backgrounds; wildtype, and Glr1, Gpx1, Gpx2, Grx1, Grx2 and Yap1 mutants leading to 14 different conditions (i.e., 2×7). We assign the capacity of each network edge using the capacity of the reactant gene. For each condition, we calculate the capacity of each gene as log(*e*_*g*_)/*κ*, where *e*_*g*_ and *κ* represent the expression level of gene *g* and capacity constant, respectively. In our experiments, we use *κ*=2. There are several main reasons behind our choice of capacity function. First, one could replace the gene expression levels with the protein abundance values. However, when the protein abundance data is not available, transcription values are often used as an indicator of the protein abundance. Second, logarithmic transformation is commonly used in studying microarray gene expression data [37] as gene expression values are highly skewed. Logarithmic transformation stabilizes the variance, compresses the range of data and makes the data more normally distributed so it allows statistics to be applied to the data.

**Fig. 4:**
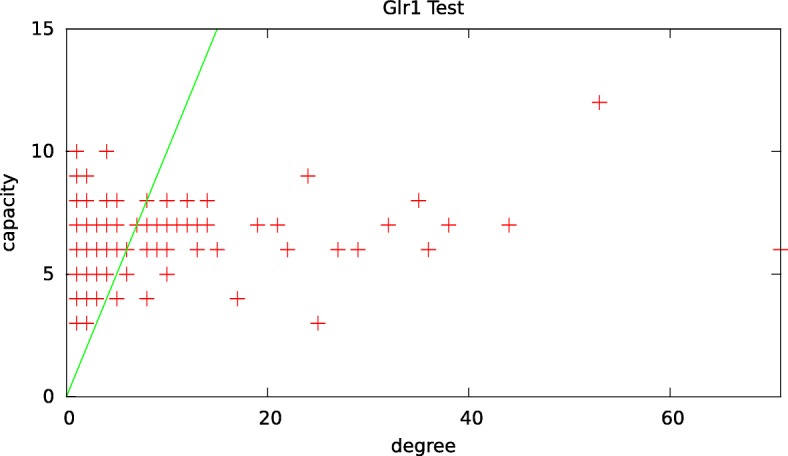
The distribution of capacity levels and degrees of reactant genes for Glr1 oxidative stress condition. The diagonal line shows the degree = capacity line

In order to observe whether there is any correlation between the topology of the genes in the interaction networks and their transcription (thus the capacity), we create a scatter plot for the distribution of capacity levels and degrees of reactant genes for each mutant and wild type under normal and oxidative stress conditions. Figure 4 presents the results for Glr1 mutant under oxidative stress condition. We observe that there is no correlation between node degree and capacity levels of genes. We observe the same pattern for all the remaining 13 conditions (results not shown).

IMPLEMENTATION AND SYSTEM DETAILS. We implement the *POMOC* algorithm in C++. We perform all the computational experiments on a Linux machine equipped with Intel core i7 processor 3.6 GHz CPU and 12GBs RAM.

### Evaluation on synthetic networks

In this section, we compare *POMOC* to motif counting with *F*_1_ and *F*_2_ measures on synthetically generated networks for varying network size, network topology and edge capacity.

#### Effects of network size

**Fig. 5:**
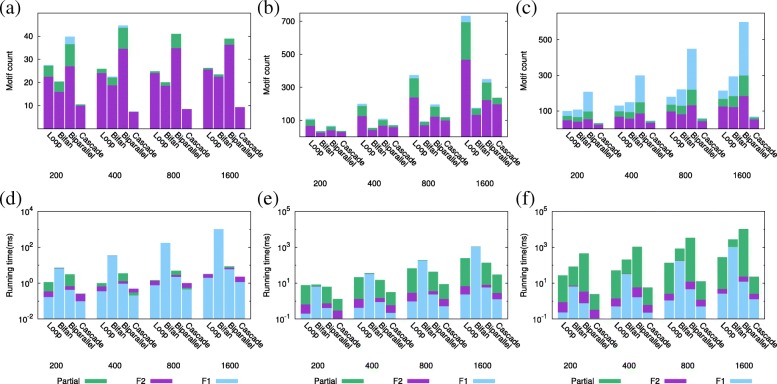
Motif count and running time on varying sizes of synthetic networks generated by (**a**, **d**) ER, (**b**, **e**) WS and (**c**, **f**) BA network topology models

We generate random networks of varying sizes (200, 400, 800 and 1600 nodes) using ER, WS and BA topology models. We set the average degree of nodes to six, and the capacity for each edge to two. For each network size and model we generate 10 networks, and report the average motif count and running time. Figure 5 reports the results.

*Effects of network size on motif count* (Fig. 5a to c). We observe that motif count using partial overlaps significantly differs from that of *F*_1_ and *F*_2_ measures. Motif counting with *F*_2_ measure finds substantially lower motif counts than our method for each motif type in all network topologies for almost all motif types. In ER networks, only biparallel motif count shows observable difference between partial overlap and *F*_1_ measure. In WS networks, feed forward loop and biparallel motifs show the highest differences. In BA networks, all motif topologies show difference; the most significant variation is observed for the biparallel motif. The *POMOC* method and motif counting with *F*_1_ measure shows the most significant difference in BA networks. This difference can be explained as follows. ER and WS models generate synthetic networks whose nodes have similar degrees leading to uniform motif distributions in these network topologies. However, BA networks contain hub nodes with high degrees, which leads to non-uniform distribution of motifs to edges. Thus all motif embeddings can be realized in ER and WS networks but not in BA without violating our edge capacity constraints.

All three motif counting methods show the least motif count in synthetic networks generated under the ER model. Motif counts in WS and BA networks are comparable, but substantially higher than those in ER networks. This difference can be explained by the fact that ER model generates networks that are more disconnected compared to WS and BA models. Our results also show that regardless of the network model and counting measure the motif count varies dramatically across different motif topologies. More importantly, the motif count distribution exhibits significant variance across different random network models (see Fig. 5a to c). While biparallel motif is observed most in ER and BA networks, feed forward loop is the most abundant motif in WS networks. Cascade motif is found least in ER and BA networks, but bifan is the least abundant motif in WS networks.

*Effects of network size on running time* (Fig. 5d to f). Our results demonstrate that counting motifs takes the most time with partial overlap constraint, and least time with *F*_1_ measure for all network sizes, topologies and motif types. This is not surprising since *F*_2_ count and partial overlap require solving the *F*_1_ count as the first step. Furthermore, since *F*_2_ count enforces identifying non-overlapping set of motifs, it eliminates the motif embeddings identified in *F*_1_ count more aggressively than the partial overlap. That said, we observe that the running time of our method is either very close to those of *F*_1_ and *F*_2_ measures or remains to be practical for all network sizes and motifs we test.

We observe that the running times for all three measures are greatly affected by the underlying network topology. Although all running times are less than 1 s for ER and WS networks, they go up to 10 s for BA networks. The second factor that influences the running time of our method is the motif topology. While our method’s running time for bifan motif is largest on ER and WS networks, that for biparallel motif is largest on BA networks. Cascade motif takes the shortest time in all three network topologies due to its smaller abundance and simple three node topology.

Three motif counting approaches show network topology dependent differences. In ER networks, only running times for feed forward loop and biparallel motifs show observable difference for the three measures. Similarly, in WS networks, running times for feed forward loop, biparallel and cascade motifs differ. The running time for our method differs from that of the *F*_2_ measure by a larger margin on BA networks for all motif types and network sizes. The most significant gap is observed for the biparallel motif. Finally, we would like to note that running time is independent of the motif count in ER and WS networks. However, there is a positive correlation between motif count and running time for BA networks; running time increases with increasing motif count.

**In summary**, our experiments suggest that motif distributions are heavily impacted by the network topology. In particular, BA networks show the most difference between *POMOC* method, and motif counting with *F*_1_ and *F*_2_ measures. Since biological networks often have similar topological characteristics as BA networks [2], we conjecture that our method will be crucial in determining the motif counts for real networks. On the other hand, our experiments also suggest that *POMOC* method is slightly slower than motif counting with *F*_1_ and *F*_2_ measures. While all three network topologies show running time differences for three motif counting approaches, the most significant difference is observed in BA networks. In all three network models, we observe that as network size increases running time increases linearly. Our method, however, scales to large networks (it runs in less than 10 s even for a network with 1600 nodes). Thus, our method is scalable to genome scale biological networks.

#### Effects of capacity

**Fig. 6:**
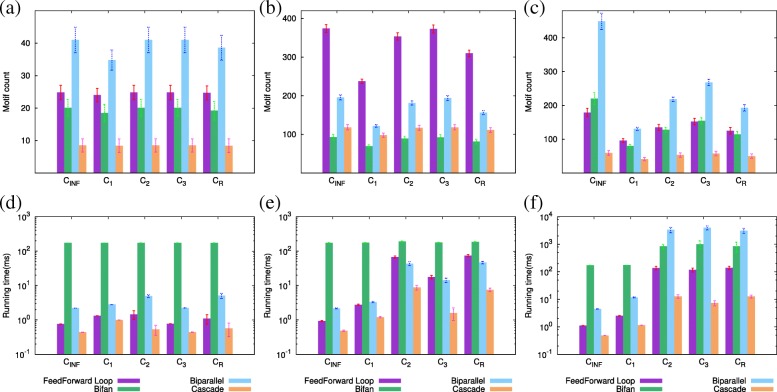
Motif count and running time on synthetic networks generated by (**a**, **d**) ER, (**b**, **e**) WS and (**c**, **f**) BA network models with varying edge capacity levels

Here, we compare the performance of our method to that of motif counting with *F*_1_ and *F*_2_ measures under varying edge capacity levels. We compare these three approaches using two metrics: motif count and running time. Similar to the previous section, we generate random networks with size 800 and average node degree 6 using ER, WS and BA network topology models. The capacity for each network edge is set using two distinct approaches. In the first approach we set capacity of all edges to 2 (*C*_2_) or 3 (*C*_3_). In the second approach, for each network edge, we randomly set capacity to an integer between 1 and 3 (*C*_*R*_). Note that motif counting with *F*_1_ and *F*_2_ measures correspond to setting the edge capacity to infinity (*C*_*INF*_) and one (*C*_1_), respectively. For each edge capacity and network topology model we generate 10 random networks, and report the average and two standard errors of motif count and running time. Figure 6 shows the results.

*Effects of capacity on motif count* (Fig. 6a to c). Our results suggest that the effects of edge capacity depends greatly on the network topology model. The motif counts for the WS and BA networks are substantially higher than that for the ER network. For the ER network, at all capacity levels, biparallel and cascade motifs are observed the most and the least, respectively. In WS networks, while feed forward loop is observed the most, bifan motif is observed the least in this network topology. In BA networks, biparallel and cascade motifs are observed the most and the least, respectively.

Next, we analyze how the motif counts change as edge capacity increases. We observe that as the capacity level increases the number of motifs found by the *POMOC* method tends to increase. This is not surprising as higher capacity values make it possible to have more motifs share the same edge without violating the capacity constraints. Motif counting with *F*_1_(*C*_*INF*_) and *F*_2_(*C*_1_) measures show the most and least number of motifs, respectively. Motif counting with varying edge capacities (*C*_*R*_) results in motif counts that are comparable to *C*_1_ and *C*_2_. Increasing edge capacity affects the motif counts in different ways for the three network topologies. In ER and WS networks, number of motifs are comparable between motif counting with *F*_1_ measure and partial motif counting with an edge capacity of 2 and 3, respectively. This suggests that for these two network topologies, a small edge capacity is enough to find all possible embeddings for each motif type. However, in BA networks, the motif counts are vastly different between motif counting with *F*_1_ measure and *POMOC* method. For this network topology, even an edge capacity of 3 is not enough to find all network motif embeddings. This observation can be explained by the fact that ER and WS models generate synthetic networks whose nodes have similar degrees. This leads to uniform motif distribution in these two network topologies with small number of motif overlaps. Thus, all motif embeddings could be found by using small edge capacities. However, the scale-free networks generated by BA model contain hub nodes with high degrees. The distribution of motifs to edges are non-uniform in these networks leading to large number of motif overlaps. Thus, all motif embeddings cannot be realized in BA networks without violating edge capacity constraints even with an edge capacity of 3–resulting in much lower motif counts as compared to the *F*_1_ measure.

*Effects of capacity on running time* (Fig. 6d to f). Our results demonstrate that our method is very fast for both fixed and variable capacity values. We observe that *POMOC* method’s running time for four motifs is affected by the network topology. The running time for all network motifs is less than one second in ER and WS networks. Although finding motifs in BA networks takes slightly longer, running time is still less than 5 s. Counting bifan and cascade motifs take the longest and the shortest time in ER and WS networks, respectively. Our algorithm shows different performance for BA networks. While bifan motif’s running time is highest in motif counting with *F*_1_ and *F*_2_ measures, biparallel motif count’s running time is highest for motif counting with edge capacity levels; *C*_2_,*C*_3_ and *C*_*R*_. For all capacity levels, cascade motif takes the shortest time in this network topology.

Running time for each motif changes similarly for ER and WS networks; in general motif counting with *C*_*R*_ and *C*_*INF*_ takes the longest and the shortest time, respectively. Interestingly, our method’s running time does not increase or decrease monotonically as edge capacity increases in these two network topologies. While running time increases from edge capacity of 1 to 2, the opposite behavior is observed as edge capacity increases from 2 to 3. Again, our algorithm shows slightly different behavior in BA networks. The running time increases as edge capacity increases from 1 to 2, but it is comparable for the capacity levels 2 and 3. In ER and WS networks, the running time does not correlate the motif counts. However, BA networks show positive association between motif count and running time.

**In summary**, our experiments suggest that BA networks will benefit from our partial motif counting method the most. Since, BA model generates scale-free networks that resemble the real biological networks, our method will potentially lead to more accurate motif counts and biological interpretations. On the other hand, our experiments also demonstrate that *POMOC* method is fast (less than 5 s) for a network of 800 nodes. Thus, we conjecture that that our method can scale to large real biological networks. Next, we test this conjecture by running our method on real networks.

### Evaluation on real networks

**Fig. 7:**
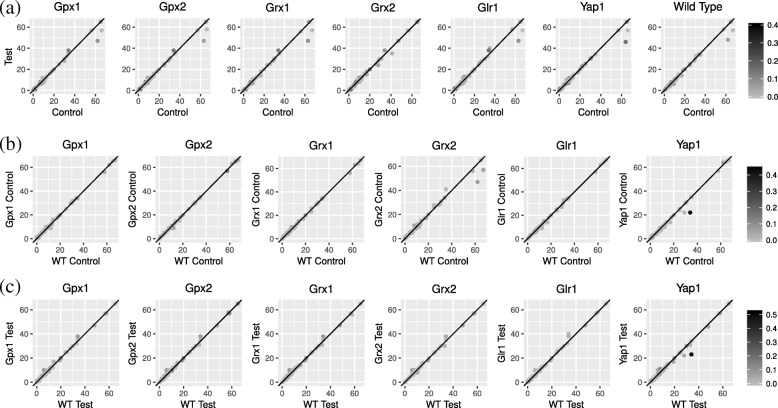
Motif counts for all reactant genes under control and oxidative stress conditions in seven genetic backgrounds (**a**), and in wild type and six genetic mutants under control (**b**) and oxidative stress (**c**) conditions

In this section, we evaluate the performance of the *POMOC* method on a *S. cerevisiae* (budding yeast) transcriptional regulatory network. In our analysis, we focus on the effects of edge capacity to motif counting in seven genetic backgrounds (wild type, and Gpx1, Gpx2, Grx1, Grx2, Glr1 and Yap1 mutants) under two experimental conditions (normal and oxidative stress). As explained above, the *POMOC* method uses gene expression levels to calculate the network edge capacities. Thus, we use our method to count motifs of the yeast transcription network for 14 edge capacity vectors. Tables 1, 2 and 3 and Fig. 7 present our results. The running times for motif counting with *F*_1_ and *F*_2_ measures are less than 1 s in all real network experiments. *POMOC* method’s running time–in average 22 s–is comparable to those of *F*_1_ and *F*_2_ measures, and remains to be practical for real biological networks.

**Table 1:** Average and standard deviation of the motif counts for four motif topologies using the *POMOC* method and, motif counting with *F*_1_ and *F*_2_ measures

	Feedforwad	Bifan	Biparallel	Cascade
*F*_1_	73	2026	11	1
*F*_2_	24	137	5	1
*POMOC*	54±0.5	426.4±7	10±0	1±0

**Table 2:** Top five genes with most publication count among the genes identified by *POMOC* but not by the *F*_2_ measure for motifs feed forward loop (FFL) and bifan

FFL	Count	Bifan	Count
CLN2	44	IDH2	111
ENO1	40	DDR2	7
ILV2	26	PEX1	7
ILV5	20	YRR1	7
INO1	16	ILV1	4

**Table 3:** Motif count variation for Abf1, Dal80, Msn2, Msn4, Skn7 and Yap1 under control and oxidative stress conditions in seven genetic backgrounds (a), and in wild type (WT) and six genetic mutants under control (b) and oxidative stress (c) conditions. The first and second digits represent whether the observed difference in edge capacity and motif count is significant (1) or not (0)

		Gpx1	Gpx2	Grx1	Grx2	Glr1	Yap1	WT
(a)	Abf1	00	00	00	01	00	01	01
	Dal80	00	00	00	10	00	00	00
	Msn2	01	01	01	00	01	01	01
	Msn4	01	01	01	00	01	11	01
	Skn7	00	00	00	00	00	00	00
	Yap1	11	11	11	01	11	00	00
		Gpx1	Gpx2	Grx1	Grx2	Glr1	Yap1	
(b)	Abf1	00	00	00	00	00	01	
	Dal80	00	00	00	00	00	00	
	Msn2	00	00	00	01	00	00	
	Msn4	00	00	00	01	00	00	
	Skn7	00	00	00	00	00	01	
	Yap1	00	00	00	00	00	11	
		Gpx1	Gpx2	Grx1	Grx2	Glr1	Yap1	
(c)	Abf1	01	01	11	01	01	01	
	Dal80	10	10	10	00	00	00	
	Msn2	00	00	00	00	00	00	
	Msn4	00	00	00	00	00	00	
	Skn7	00	00	00	00	00	01	
	Yap1	01	01	01	01	01	11	

#### Average motif counts

Here, we compare the average motif counts for four motif topologies over the 14 input networks using the *POMOC* method and, motif counting with *F*_1_ and *F*_2_ measures. Table 1 reports the mean and standard deviation of motif count for each counting approach. Note that *F*_1_ and *F*_2_ measures do not utilize edge capacity, and thus, their motif counts do not change under varying edge capacity vectors. Our results show that *POMOC* finds substantially different number of motifs than that with *F*_1_ and *F*_2_ measures. We observe that there is a massive variation in the abundance of different motif topologies from only one up to over two thousand. There is only one instance of cascade. Thus, this motif instance occurs in all three measures as it has no other instance to overlap with. More importantly, partially overlapping motif counting varies greatly in the interval defined by *F*_1_ and *F*_2_ measures for different motif topologies. For instance, for biparallel, it is closer to *F*_1_ measure, while it is closer to *F*_2_ measure for bifan, and equally distant to *F*_1_ and *F*_2_ measures for feed forward loop. This demonstrates that the amount of overlap among different embeddings of a motif depends not only on the network topology but also the motif topology. Similar to Milo et al. (2002) [8], we find that bifan and feed forward motifs are the most abundant motif types in this network. These two motif topologies also show small variation across the 14 networks for the *POMOC* method. Finally, our results show that the motif count distribution in yeast network differs substantially from that of all three synthetic network models we test (see Fig. 5a to c). Biparallel motif is the most abundant in ER and BA networks, and feed forward is the most abundant in WS network. On the other hand, bifan is the most abundant motif in yeast network with a huge margin.

**In summary**, our partial motif counting approach finds vastly different motif counts than *F*_1_ and *F*_2_ measures. Since real biological interactions are resource limited, leading to varying interaction (edge) capacities, we hypothesize that our method provides a more accurate approach to count network motifs, and to decipher dynamics of biological systems under varying conditions. Our method shows little variation in motif counts on the yeast transcription network (see the standard deviation values in Table 1), which suggests that variation in gene expression does not substantially change the edge capacity levels for the yeast network. That said, by focusing on the edges, which lead to such minor variation can reveal subtle differences between the impact of different cell states. Next, we further investigate the difference among three measures, *F*_1_,*F*_2_ and *POMOC*.

#### Comparison with *F*_1_ and *F*_2_ measures

Here, we focus on the genes that exhibit differences in motif count among *F*_1_ measure, *F*_2_ measure and *POMOC*. To do this, for each frequency measure, we obtain the set of genes which are included in the resulting embedding set for each genetic background under oxidative stress condition. Notice that the gene sets identified by *F*_1_ and *F*_2_ measures are same across all conditions. They however may differ for *POMOC*. We represent the gene set for *F*_1_ and *F*_2_ measures with $A_{F_{1}}, A_{F_{2}}$ respectively. For each genetic background, say the *i*th background, we represent the gene set identified by *POMOC* with $A_{P_{i}}$. Notice that, for all *i*, the gene set $A_{P_{i}}$ is always a subset of $A_{F_{1}}$. We first investigate the difference among genes identified by *F*_1_ measure and *POMOC* respectively. To do this, we do the literature analysis on genes included by motif instances. For each gene $\phantom {\dot {i}\!}g_{j} \in A_{P_{i}}$, we count the number of publications containing gene *g*_*j*_ and each of the three keywords ‘oxidative’, ‘respiration’ and ‘fermentation’ in PubMed. For each gene $\phantom {\dot {i}\!}g_{j} \in A_{F_{1}}$, we also count the motif embeddings containing *g*_*j*_ identified by *F*_1_ measure and *POMOC* respectively. Here, we calculate the latter as the mean value of the number of embeddings containing *g*_*i*_ for all seven genetic backgrounds under oxidative stress conditions. Then we calculate the Spearman correlation between publication count and gene specific motif count generated by *F*_1_ and *POMOC* respectively. We observe that the gene set identified by *POMOC* exhibits much higher correlation with publication count (feed forward loop: correlation = 0.265, bifan: correlation = 0.248) than that by *F*_1_ measure (feed forward loop: correlation = 0.158, bifan: correlation = 0.007), which implies that our method has the potential to filter genes that do not exhibit significant publication evidence. To test our hypothesis, we collect all genes that belong to $A_{F_{1}}$ but not *POMOC*, that is $\bigcup _{i}{A_{F_{1}} \backslash A_{P_{i}}}$. We observe that these genes contribute to limited publication counts compared to the number of these genes especially for feed forward loop (i.e., feed forward loop: number of genes percentage = 25.26%, publication count percentage = 4.86%, bifan: number of genes percentage = 1.88%, publication count percentage = 0.24%). Thus, the genes found by *F*_1_ but not by *POMOC* have very low publication evidence to the oxidative stress.

Next, we investigate the difference between *F*_2_ measure and *POMOC*. Similar with the comparison between *F*_1_ measure and *POMOC*, we collect all genes that belong to *POMOC* but not in $A_{F_{2}}$, that is $\bigcup _{i}{A_{P_{i}} \backslash A_{F_{2}}}$. Notice that the gene set identified by *POMOC* is often larger than that by *F*_2_ measure (but not necessarily a superset). Table 2 presents the top five genes with the largest publication count for feed forward loop and bifan. We observe that there is substantial publication evidence for these genes. For example, Mitochondrial NAD^+^-specific isocitrate dehydrogenases (IDHs) are key regulators of flux through biosynthetic and oxidative pathways in response to cellular energy levels [38]. One lysosomal protease which when mutated can cause juvenile onset neuronal ceroid lipofuscinoses (NCL) is encoded in CLN2, a serine tripeptidyl protease with no obvious yeast orthologue [39]. Thus, *POMOC* has the potential to discover significant genes which are missed using *F*_2_. Next, we analyze the association between motif count and edge capacity focusing on individual genes.

#### Gene specific motif counts

Here, we take a closer look into the network contents and analyze the distribution of motif embeddings to individual genes. To do that, we first find the set of partially overlapping motif embeddings for each of the 14 genetic background and experimental condition combination. For each gene in each network, then, we count the number of motifs containing that gene. Figure 7 plots these counts for all reactant genes that we use to assign the edge capacities in the *S. cerevisiae* transcriptional regulatory network. In what follows, we discuss the motif count differences and effects of the edge capacity levels to motif counting in seven genetic backgrounds under control and oxidative stress conditions.

**Effects of experimental condition to motif count.** Our results demonstrate that most of the genes exhibit similar motif counts under varying genetic backgrounds and growth conditions. We observe more genes away from the *y*=*x* line in control *vs.* oxidative stress conditions (Fig. 5a) than wild type *vs.* mutant genetic backgrounds (Fig. 5b and c). This suggests that oxidative stress is more disruptive to the organization and abundance of motifs in yeast network than mutations of individual genes.

To understand our results more clearly, from now on, we focus on the analysis of the six reactant genes; Abf1, Dal80, Msn2, Msn4, Skn7 and Yap1. Among these, Msn2, Msn4, Skn7 and Yap1 play key roles in controlling the transcription of oxidative stress response genes [40–44]. Dal80 is implicated in nitrogen depletion and amino acid starvation stress responses [45, 46]. Similarly, Abf1 is involved in nutritional stress response [47]. Table 3 reports how changes in the edge capacity levels affect the number of motifs these six genes participate. For this analysis, first we divide the edge capacity and motif count variations to two groups: significant or non-significant. We designate any variation that is more than 10% as significant. We then divide all reactant genes to four groups (00, 01, 10 and 11). Here, the first digit represents whether observed variations in edge capacity is significant (1) or not (0), respectively. Second digit denotes whether the deviation in motif count is significant.

First, we focus on motif count variation in seven genetic backgrounds under control and oxidative stress conditions (Fig. 5a and second digits in Table 3a). Our analysis shows that while most of the reactant genes including Dal80 and Skn7 have similar motif counts, some genes such as Msn2, Msn4 and Yap1 show substantially different motif counts. We observe that motif count differences are highly dependent on genetic background: Msn2 and Msn4 show different motif counts in all genetic backgrounds except Grx2 mutant; Yap1 shows different motif counts only in Gpx1, Gpx2, Grx1, Grx2 and Glr1 mutants; Abf1 shows different motif counts only in wild type and, Grx2 and Yap1 mutants.

Motif counts show small variation between wild type and mutant conditions under control and oxidative stress conditions (Fig. 5b-c and and second digits in Table 3b-c). Our results suggest that genetic mutants show similar gene expression levels to wild type, which leads to comparable edge capacity levels and motif counts. Under control conditions: Msn2 and Msn4 show motif count variation in Grx2 mutant; Yap1, Skn7 and Abf1 show motif count variation in Yap1 mutant. We observe slightly different behavior under oxidative stress conditions: Yap1 and Abf1 show motif count variation in all genetic backgrounds; Skn7 shows motif count variation in Yap1 mutant. Msn2, Msn4 and Dal80 show similar motif counts under oxidative stress in all genetic backgrounds.

**Effects of edge capacity to motif count.** Here, we analyze how an individual gene’s edge capacities affect the number of motifs it participates. Figure 7 shows that while genes with highly different edge capacity (darker circles) might have no motif count variation (circles on the *y*=*x* line); genes with similar edge capacity (lighter circles) might have motif count variation (circles off the *y*=*x* line). This implies that variation in edge capacity levels is not a sufficient condition for alterations in motif count. Also small variations in edge capacities may lead to substantial changes in motif counts. We will further analyze these observations on six stress response genes. The two digits in Table 3 reports the dependency between edge capacity and motif counts.

First, we report how oxidative stress affects motif counts. We observe that although oxidative stress leads to edge capacity variation for many genes, most of the genes show similar motif counts under control and oxidative stress conditions in seven genetic backgrounds. Msn2 and Msn4 show small edge capacity variation, but high motif variation in all genetic backgrounds except Grx2 mutant (Table 3a). Abf1 shows similar edge capacity in all genetic backgrounds, however, it shows different motif counts in wild type and, Grx2 and Yap1 mutants. In Grx2 mutant, Dal80 shows edge capacity variation, but similar motif counts. For Yap1 we see a more complex story. It shows edge capacity variation in Gpx1, Gpx2, Grx1 and Glr1 mutants, which leads to different motif counts. However, while Yap1 shows similar edge capacity levels in wild type, and Grx2 and Yap1 mutants, it has different motif counts in Grx2 mutant.

In almost all genetic backgrounds, majority of the genes do not show edge capacity variation between wild type and mutant genetic backgrounds under control condition (Table 3b). Yap1 shows edge capacity and motif count variation in Yap1 mutant. Msn2 and Msn4 show similar edge capacity but different motif counts in Grx2 mutant compared with wild type. Similarly, Abf1 and Skn7 show motif count variation in Yap1 mutant. Oxidative stress leads to slightly more edge capacity variation compared to control (Table 3c). Yap1 and Abf1 show edge capacity and motif count variation in Yap1 and Grx1 mutants compared with wild type, respectively. Dal80 shows edge capacity variation in Gpx1, Gpx2 and Grx1 mutants, however, it has similar motif counts. Despite lack of edge capacity variation, Yap1 shows different motif counts between wild type and, Gpx1 Gpx2, Grx1, Grx2 and Glr1 mutants. Similarly, Abf1 does not show edge capacity variation in Gpx1, Gpx2, Grx2, Glr1 and Yap1 mutants, but, it has different motif counts. Finally, Skn7 shows motif count variation in Yap1 mutant although having similar edge capacity.

**In summary**, partial motif counting approach finds substantially different motif counts than that of *F*_1_ and *F*_2_ measures. However, the changes in edge capacity is not enough to explain the observed motif count differences for individual genes. While some genes show different motif counts due to edge capacity changes, most genes have the same motif count. There are also genes that show similar edge capacity levels but different motif counts. For these genes, the changes in motif counts could be possibly explained by the edge capacity changes of its neighboring genes.

## Discussion and Conclusions

Motif counting in biological networks is an important tool to decipher the topology of biological networks and its function. Existing motif counting approaches either count all or non-overlapping instances of a given motif. This results in either over- or under-estimation of the motif counts, since biological reactions are constrained by many factors such as concentration levels of reactant molecules. In this paper, we introduced a novel frequency measure which considers the abundance of the interacting molecules. It allows partially overlap between different embeddings based on edge capacities. This problem is fundamentally different from the problem of finding weighted network motifs which also incorporates the edge information. This is because the edge weights in weighted network is used to identify if an embedding has strong strength. And the resulting embeddings can be counted using both *F*_1_ and *F*_2_ measure. In our problem, the edge information however is used to specify at most how many embeddings can share this edge, leading to motif count different from both *F*_1_ and *F*_2_ measure. Moreover, the resulting embeddings in our problem do not contain weight.

To address this problem, we presented a novel motif counting method, *POMOC*, based on edge capacities of a given network. Our experiments on both synthetic and real networks demonstrate that motif count using our method significantly differs from the existing motif counting approaches, and our approach extends to large-scale biological networks in practical time. Application of our method to the *S. cerevisiae* transcriptional regulatory networks demonstrates that the *POMOC* method reveals topological differences of biological networks under different genetic backgrounds and experimental conditions.

## Abbreviations

BA: Barabasi-albert
ER: Erdos-renyi
FFL: Feed forward loop
IDHs: Isocitrate dehydrogenases
MIS: Maximum independent set
NCL: Neuronal ceroid lipofuscinoses
POMOC: Partially overlapping motif counting
WS: Watts-strogatz
WT: Wild type
